# The Mediterranean and Black Sea Fisheries at Risk from Overexploitation

**DOI:** 10.1371/journal.pone.0121188

**Published:** 2015-03-20

**Authors:** Athanassios C. Tsikliras, Anny Dinouli, Vasileios-Zikos Tsiros, Eleni Tsalkou

**Affiliations:** Laboratory of Ichthyology, Department of Zoology, School of Biology, Aristotle University of Thessaloniki, Thessaloniki, Greece; Institute of Marine Research, NORWAY

## Abstract

The status of the Mediterranean and Black Sea fisheries was evaluated for the period 1970-2010 on a subarea basis, using various indicators including the temporal variability of total landings, the number of recorded stocks, the mean trophic level of the catch, the fishing-in-balance index and the catch-based method of stock classification. All indicators confirmed that the fisheries resources of the Mediterranean and Black Sea are at risk from overexploitation. The pattern of exploitation and the state of stocks differed among the western (W), central (C) and eastern (E) Mediterranean subareas and the Black Sea (BS), with the E Mediterranean and BS fisheries being in a worst shape. Indeed, in the E Mediterranean and the BS, total landings, mean trophic level of the catch and fishing-in-balance index were declining, the cumulative percentage of overexploited and collapsed stocks was higher, and the percentage of developing stocks was lower, compared to the W and C Mediterranean. Our results confirm the need for detailed and extensive stock assessments across species that will eventually lead to stocks recovering through conservation and management measures.

## Introduction

Overexploitation of marine fisheries resources is either known or suspected for almost all the commercial fish stocks of the world [1]. This is the general rule, which holds globally. There are, however, a few exceptions of healthy fisheries, as a result of good management practices [2], that are justifying the rule. The issue of fisheries overexploitation at a global scale has been realized at least since 2001 [3], although there have been some earlier stock depletions that received extended news coverage, such as that of cod *Gadus morhua* in Canada in 1992 [4]. In the Mediterranean and the Black Seas (FAO Major Fishing Area 37), overexploitation has been recently shown to occur for the entire area [5,6] and for specific regions (e.g. Greek Seas [7]). Several Mediterranean stocks have been reported overexploited based on data from landings [5,7], scientific surveys [8], or stock assessments [9]. As European fisheries are no exception to the rule, much of the EU fisheries legislation actually aims to tackle with the problem of overexploitation (e.g. Council Regulations 1967/2006, 643/2007, 1224/2009, 404/2011), including the relatively recent Marine Strategy Framework Directive through its Descriptor 3 (Commission Decision 2010/477/EU) [10]. However, in parallel with global economy, management regulations are not really implemented by all parties involved, especially by those who profit for not respecting the commons; this situation may lead to ecosystem crises [11].

Long-term worries about the state of stocks explain why fishery-independent stock assessments have been carried out for the past twenty years over the northern part of the Mediterranean basin [9,12], in parallel with extended monitoring of the landings that is anticipated through the EU Fisheries Data Collection Framework (Council Regulation 1999/2008). In the Mediterranean, regular scientific stock assessments are mainly carried out within two international bodies, the General Fisheries Commission for the Mediterranean (GFCM) of the Food and Agricultural Organization (FAO) and the International Commission for the Conservation of Atlantic Tunas (ICCAT), which is an inter-governmental fishery organization responsible for the conservation of tunas and tuna-like species in the Atlantic Ocean and the Mediterranean Sea. The Scientific Technical and Economic Committee for Fisheries (STECF) of the EU [12] also conducts assessments for the stocks exploited by the EU countries of the northern Mediterranean coastline. Thus, data collection, stock assessment and management of Mediterranean fisheries are gradually improving, even though there are still areas with no or inadequate fisheries information (e.g. eastern Mediterranean). Management information and guidelines resulting from stock assessments and scientific surveys, can now be delivered to the involved parties and the public at a finer geographical scale and more timely [9].

Trophodynamic indicators have been widely used in marine ecology [13] and trophic level based indicators are valuable for identifying the expansion or contraction of fisheries [14], as well as for quantifying the effects of fishing on the trophic structure of food webs [8] and the function of marine ecosystems [15], including loss of biodiversity [14]. Among the several indicators that have been used, the trophic level of the catch and the fishing-in-balance index have been extensively tested [13] and have been proven robust in tracking fishing effects [16], and assessing ecosystem changes [17]. Therefore, they are considered promising indicators for ecosystem management [13].

Mediterranean fisheries are multispecies and generally lack large monospecific stocks compared to the open ocean [18]. Consequently, the acknowledged disadvantages of stock assessments and scientific surveys, i.e., that they include a limited number of stocks compared to the total (around 25% in terms of landed biomass, less than 10% in terms of stock numbers) and are conducted at irregular basis [19], are valid for the area. Indeed, the larger proportion of landed biomass in the Mediterranean comes from data-deficient fisheries [20], i.e., stocks that have never been properly assessed, a proportion that has been estimated at around 80% [21].

The official data on fisheries catches (equivalent to landings for the purposes of this work) has been widely used to examine the state of the exploited marine resources and to test large-scale ecological hypotheses. In particular, the catch-based method of stock classification to exploitation categories has been extensively used to assess the status of fisheries globally or on ecosystem basis [5,7,22–26]. After the taxonomic, quantitative and spatial improvements through the completion of the global reconstruction of catches [27], the validity of catches will no longer be questioned. However, since there is a global debate on whether data from catches or from surveys and stock assessments are more suitable for examining the exploitation status of marine fish stocks [19], data from scientific surveys and stock assessments, when available, could be used complementary to catch data.

Earlier research on the Mediterranean and Black Sea fisheries report that 78% of the stocks are fully exploited in the Mediterranean, and 85% of the stocks are overexploited in the Black Sea [28,29]. Overexploitation was evident since the 1950s, when about 60% of the Mediterranean and Black Sea stocks were reported as being fully exploited and 40% as overexploited [30]. When the catch-based method was applied on the combined landings of Mediterranean and Black Sea, over 80% of the Mediterranean stocks were described as fully exploited [31] and around 90% of the Black Sea stocks as collapsed, in 2004 [32]. Recently, using the same method on the same area, the cumulative percentage of collapsed and overexploited stocks exceeded 60% [5]. Based on scientific stocks assessments, 85% of the assessed stocks have been reported as being overfished compared to their maximum sustainable yield value [9]. According to the most recent work, the exploitation rate in the Mediterranean has been steadily increasing and selectivity deteriorating; thus leading to shrinkage of fish stocks [6]. Finally, a low rate decline in the mean trophic level of the catches has been reported for the Mediterranean and Black Seas [33].

In our previous work on the Mediterranean and the Black Sea fisheries [5], the stocks of the two seas were combined (as the FAO Area 37), for comparability purposes with previously published items. However, because of the different environmental and fishing properties of the two seas [28,34], their subareas and subdivisions [35,36], a further investigation of their fisheries status was necessary at a finer geographical scale, i.e., on a subarea basis.

The aim of the present work was to assess the exploitation status of the Mediterranean and Black Sea stocks for the period 1970–2010, using various indicators including the temporal variability of total landings, the number of recorded stocks, the mean trophic level of the catch, the fishing-in-balance index and the catch-based method of stock classification. These indicators were separately applied to the western, central and eastern Mediterranean Sea, thus including areas where primary data, such as fisheries independent data and stock assessment information, are lacking. Contrary to recent studies on the Mediterranean fisheries [5,9], which considered only the stocks that had been previously assessed and form a minor proportion of the commercially exploited stocks, in the present work, we considered all stocks for which landings data were available.

## Materials and Methods

**Fig 1:**
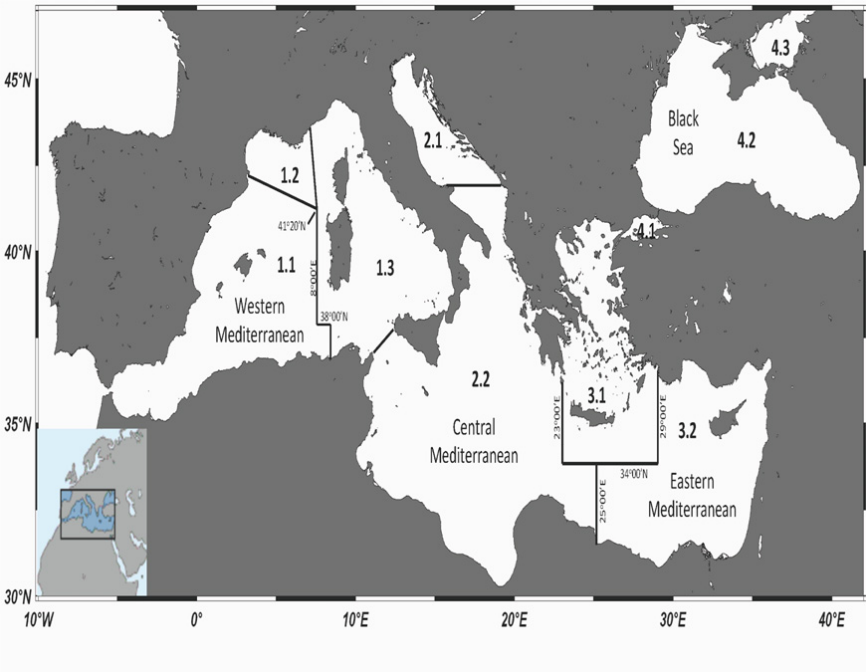
A map of the Mediterranean and the Black Sea (FAO Major Fishing Area 37) subareas (western Mediterranean, central Mediterranean, eastern Mediterranean, Black Sea) and their subdivisions (1.1: Balearic; 1.2: Gulf of Lions; 1.3: Sardinia; 2.1: Adriatic Sea; 2.2: Ionian Sea; 3.1: Aegean Sea; 3.2: Levantine Sea; 4.1: Marmara Sea; 4.2: Black Sea, proper; 4.3: Azov Sea) according to the GFCM division. Underlying map was created using Ocean Data View Software.

The annual catches of the Mediterranean and Black Sea fisheries stocks have been recorded since 1970 by the General Fisheries Commission for the Mediterranean (GFCM) [37]. The GFCM dataset differs from that of the FAO in terms of area and species aggregation. As opposed to the FAO grouping of the entire Mediterranean and the Black Seas into a single area (FAO Major Fishing Area 37), in the GFCM dataset, the Mediterranean and the Black Sea are being further divided into 4 fishing subareas (Mediterranean Sea: western, W; central, C; eastern, E; and Black Sea: BS) and 10 fishing subdivisions (Fig. 1). The GFCM data refer to the legal and reported large- and small-scale fisheries catches (excluding discarded catch, illegal, unreported, recreational, subsistence and sport fishing) expressed as live weight equivalent of landings. For the present analysis, catch statistics were extracted from the GFCM dataset, which is available as a regional package in FishStat J, for the period 1970–2010, for 2636 records (= stocks, defined here as species-area-country combinations) [37].

Two trophodynamic indicators, the mean weighted trophic level of the catch (mTLc) and the fishing-in-balance (FiB) index, that have been widely used to assess the effect of fisheries on ecosystems [38], were explored. The mean weighted trophic level of the catch (mTLc) for each year (k), was calculated annually per area according to the formula [33]:

$$mTLc=\frac{\sum_{i=1}^{m}\left(TL_{i}\times Y_{ik}\right)}{\sum_{i=1}^{m}\left(Y_{ik}\right)}$$ (1)

where Y_i_ refers to the catches of a species (or group of species) i, and TL is the corresponding trophic level. The trophic levels of each species were taken from FishBase [39] and SeaLifeBase [40].

The fishing-in-balance index (FiB) of the catch for each year (k) was calculated annually per area as [41]:

$$FiB_{k}=log_{10}\left[Y_{k}{\left(\frac{1}{TE}\right)}^{mTLC_{k}}\right]-log_{10}\left[Y_{0}{\left(\frac{1}{TE}\right)}^{mTLC_{0}}\right]$$ (2)

where Y refers to the total catches in year k, mTLc is the mean weighted trophic level of the catch, TE is the mean energy-transfer efficiency between trophic levels that is assumed to be 0.1 [14,41], and 0 refers to the first year in a time-series that is used as a baseline. In the present dataset, the first year of the time-series, 1970, was set as a baseline. FiB equals to 0 for the first year of the series and remains rather stable when trophic level and catches change in opposite directions. Increasing FiB values indicate geographic or bathymetric expansion of fisheries, while decreasing FiB values indicate contraction [41].

The exploitation status of fisheries (1970–2010) was classified into one of the following five categories, according to the catch-based stock classification method [26,30]: developing, fully exploited, overexploited, collapsed, and recovering of a previously collapsed stock (Table 1). The “undeveloped” category was incorporated in “developing”. The classification is based on the relationship between the catches (C_Y_) of a given year (Y_C_) compared to the year (Y_Cmax_) of historical maximum catch (C_MAX_) (Table 1). Stocks with maximum catches appearing in the most recent year of the time series were designated as “developing” [26].

**Table 1:** The five categories (developing, fully exploited, overexploited, collapsed and recovering of a previously collapsed stock) of stock exploitation according to the catch-based method, which is based on the relationship between the catches (C_Y_) of a given year (Y_C_) compared to the year (Y_Cmax_) of historical maximum catch (C_MAX_).

Exploitation status	Criteria
Developing	Y_C_<Y_Cmax_ and 0.1C_MAX_<C_Y_<0.5C_MAX_
Fully exploited	C_Y_>0.5C_MAX_
Overexploited	Y_C_>Y_Cmax_ and 0.1C_MAX_<C_Y_<0.5C_MAX_
Collapsed	Y_C_>Y_Cmax_ and C_Y_<0.1C_MAX_
Recovering of a previously collapsed stock	Y_C_>Y_Cmax_ and 0.1C_MAX_<C_Y_

The method was applied on an annual basis to the stocks of each subarea (n = 2207 for the last year of the time series, 2010) and to the stocks of each subdivision (n = 1707 for the last year of the time series, 2010). The method was then separately applied to those stocks whose catches are available for 40 consecutive years (n = 802 for the last year of the time series, 2010). The latter analysis was included because it has been shown that the addition of new records (either because of disaggregation of higher taxonomic groups or because of exploitation of new species/stocks) in the dataset, may introduce biases in favour of developing stocks [5,7]. The number of records varies among analyses because several records were only available for non-consecutive years, some were collected from unknown or unspecified subareas and some from the entire Mediterranean Sea, such as the highly migratory bluefin tuna *Thunnus thynnus* [37].

Finally, the results and recommendations of the stock assessments routinely performed by the STECF and GFCM were retrieved from the published reports [42–46] that refer only to the Mediterranean Sea. Overall, 189 records were accessed (STECF: 18 species, 78 stocks; GFCM: 25 species, 111 stocks) from which information on current fishing mortality (F) and fishing mortality level able to provide maximum sustainable yield (F_MSY_) was extracted (for details on stock assessment methodology, assumptions, limitations and approximations see [9]). MSY is defined as the highest yield (= catch) that can be taken from a stock under existing environmental conditions, and F_MSY_ is the fishing mortality that allows stock exploitation at MSY [47,48]. The F/F_MSY_ ratio, which indicates sustainable exploitation when lower or close to 1 and unsustainable when diverging to high values [47,49], was then compared with the ratio Y_2010_/Y_MAX_ (where Y_2010_ is the latest catch and Y_MAX_ is the highest catch in the catch time series), which shows the exploitation status of the corresponding stocks according to the catch-based method. A paired match was possible for 97 stocks, either because of multiple assessments of the same stock (when multiple assessments were available for a stock, the most recent one was used) or because of unavailability of Y_2010_/Y_MAX_ records.

## Results

### Number of records

**Fig 2:**
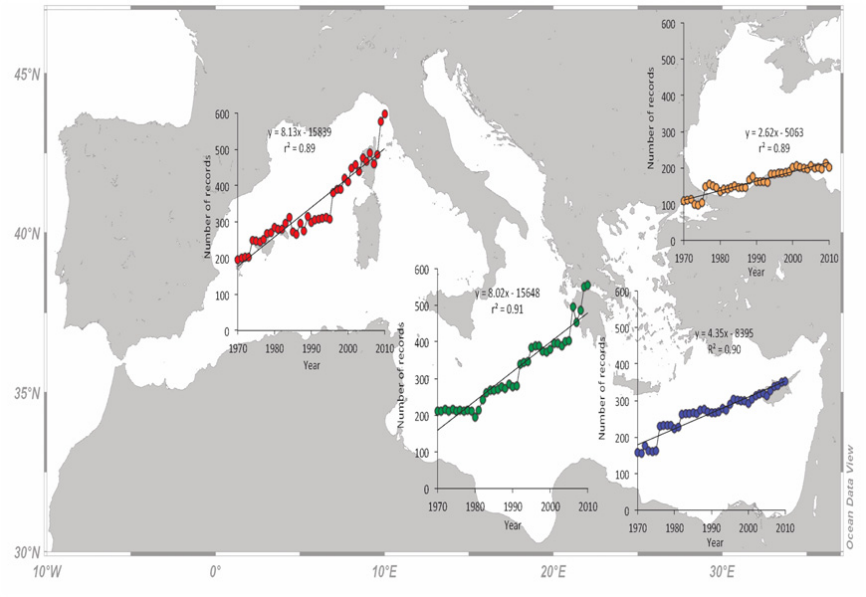
The number of stocks recorded per year for the western (red dots), central (green dots), eastern (blue dots) Mediterranean subareas and the Black Sea (orange dots) for the period 1970 to 2010.

The number of recorded fish stocks in the W and C Mediterranean increased linearly (r^2^>0.88 in both areas) from around 200 in 1970 to around 600 in 2010 (Fig. 2). A linear increase was also apparent for the E Mediterranean (r^2^ = 0.90) and the Black Sea (r^2^ = 0.89), but with fewer records (from around 150 in 1970 to around 350 in 2010 in E Mediterranean and from around 100 in 1970 to around 200 in 2010 in the Black Sea) (Fig. 2). The rate of increase for the entire study period was around 80 records per decade (i.e., the catches of 80 new stocks were added or separately recorded) for the W and C Mediterranean and lower for the E Mediterranean (44 records per decade) and the Black Sea (26 records per decade).

### Total catches

**Fig 3:**
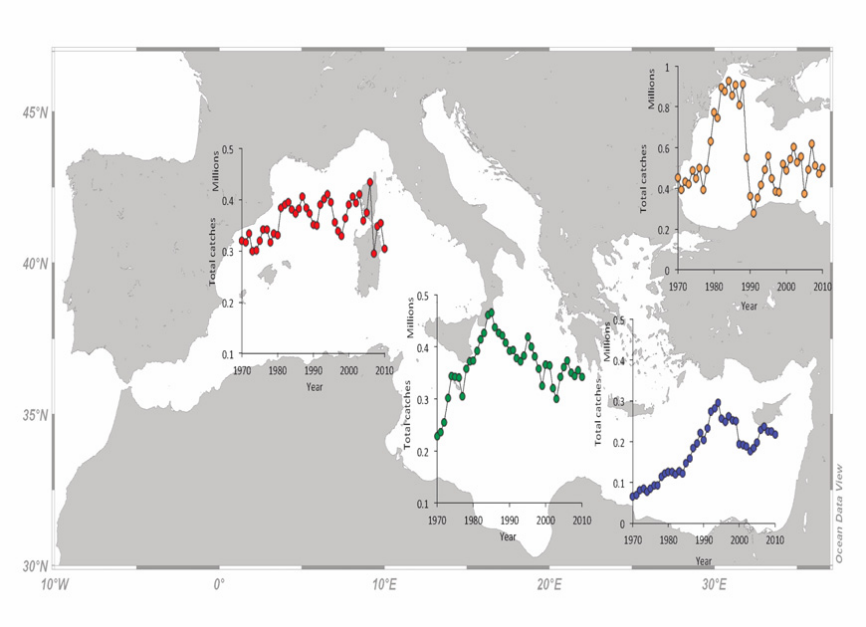
The combined marine catches (in metric tons) of fishes, crustacean and cephalopods per year for the western (red dots), central (green dots), eastern (blue dots) Mediterranean subareas and the Black Sea (orange dots) for the period 1970 to 2010.

Total catches in the W Mediterranean fluctuated around 350 000 t with 304 000 t landed in 2010 (minimum catch was 295 000 t in 2007 and maximum catch was 433 467 t in 2006) and, overall, do not show any increasing or declining trend (Fig. 3). In C Mediterranean, total catches increased rapidly from 228 400 t in 1970 to 466 218 t in 1984, then declined and fluctuate around 350 000 t for the last two decades (the catch of 2010 was 342 083 t) (Fig. 3). A similar trend was observed for the E Mediterranean but total catches were lower, the increase was smoother and lasted longer (from 66 318 t in 1970 to 295 870 t in 1994). Since 1994, total catches in E Mediterranean fluctuate around 200 000 t, with catches of 216 804 t reported in 2010 (Fig. 3). The Black Sea total catches peaked between 1980 and 1988, with values exceeding 800 000 t per year. For the periods before (1970–1979) and after that peak (1989 onwards), total catches in the Black Sea fluctuated around 450 000 t, with 501 241 t reported in 2010 (Fig. 3).

### Mean trophic level of the catch (mTLc) and fishing-in-balance (FiB) index

**Fig 4:**
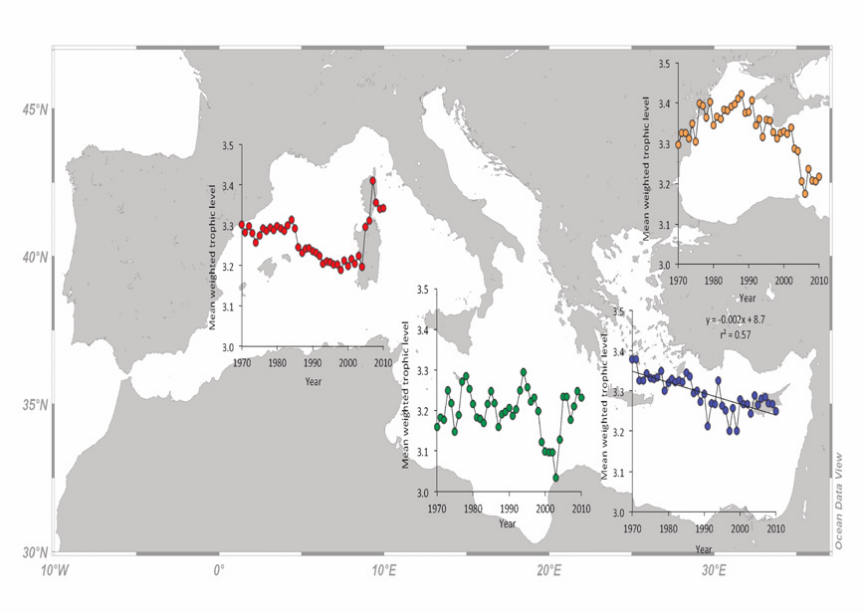
The mean trophic level of the catch (mTLc) per year for the western (red dots), central (green dots), eastern (blue dots) Mediterranean subareas and the Black Sea (orange dots) for the period 1970 to 2010.

There was a clear and steady decline of mTLc in the W Mediterranean for the period 1970–2004, from around 3.30 (1970–1985) to around 3.20 (1986–2004), at a rate of 0.03 per decade. Since 2005, mTLc increased again to around 3.35, with an irregular high peak in 2007 (Fig. 4). In the C Mediterranean, mTLc fluctuated around 3.20 for the entire period, with a five year exception (1999–2004) of lower mTLc values at around 3.10 (Fig. 4). The declining trend of mTLc in the E Mediterranean is clear and consistent throughout the study period. The rate of mTLc decline is 0.03 per decade, from 3.38 in 1970 to 3.25 in 2010 (Fig. 4). A quicker declining trend of the mTLc is also observed in the Black Sea since 1988, following an initial increase from 1970 to 1987. The rate of mTLc decline (1988–2010) is 0.09 per decade, from 3.42 in 1988 to 3.22 in 2010 (Fig. 4).

**Fig 5:**
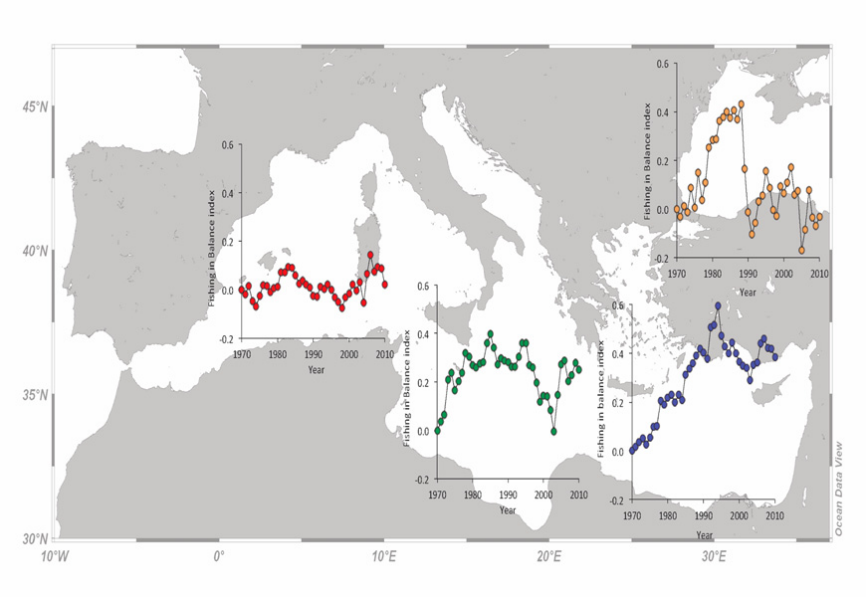
The Fishing-in-Balance (FiB) index per year for the western (red dots), central (green dots), eastern (blue dots) Mediterranean subareas and the Black Sea (orange dots) for the period 1970 to 2010.

Given the rather stable catches, the FiB index in the W Mediterranean followed a similar pattern with the mTLc, with elevated values since 2005 (Fig. 5). Similarly, in the C Mediterranean, where mTLc was rather stable, the fluctuation of FiB was determined by the catches. It was initially increasing from 1970 to 1985, then declined until the mid 2000s before rising up again (Fig. 5). In the E Mediterranean, FiB rapidly increased from 1970 to its highest value in 1994 (FiB = 0.59 in 1994) and then declined to around 0.38 in 2010 (Fig. 5). In the Black Sea, an initial increase of FiB from 1970 to its highest value in 1988 (FiB = 0.43 in 1988) was followed by a rapid decline and stabilization to values around 0 (Fig. 5).

### Exploitation status according to the catch-based method

**Fig 6:**
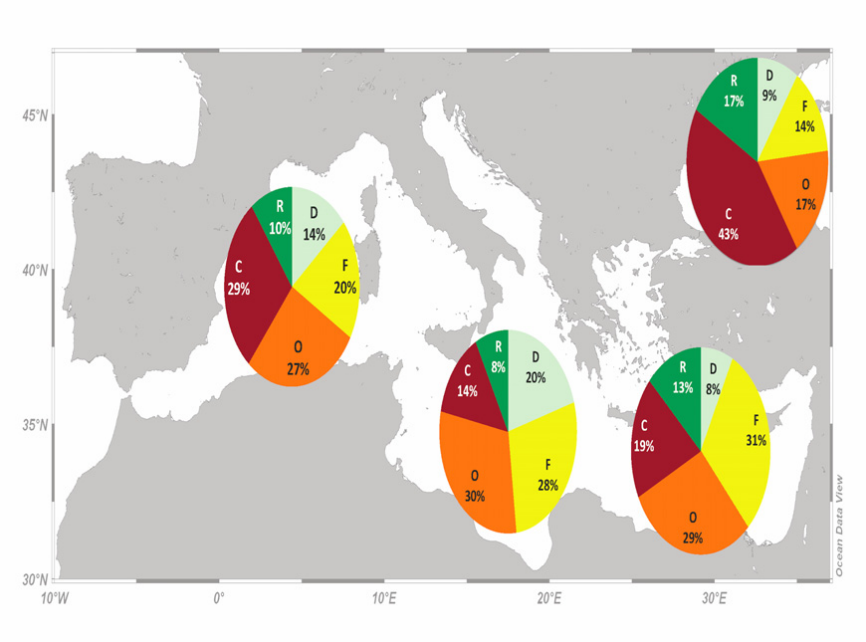
The status in 2010 of fisheries resources according to the catch-based method, for the western, central, eastern Mediterranean fishing subarea and the Black Sea for the period 1970 to 2010 (D: developing; F: fully exploited; O: overexploited; C: collapsed; R: recovering).

Based on the year and quantity of the historically maximum catch, which varied among the stocks included in the analysis, in 2010, the cumulative percentage of overexploited and collapsed stocks was 60% for the Black Sea, 56% for the W, 48% for the E, and 44% for the C Mediterranean (Fig. 6). In contrast, the cumulative percentage of developing and recovering stocks was higher in the C Mediterranean (28%) and the Black Sea (26%) compared to the W (24%) and E Mediterranean (21%) (Fig. 6).

**Fig 7:**
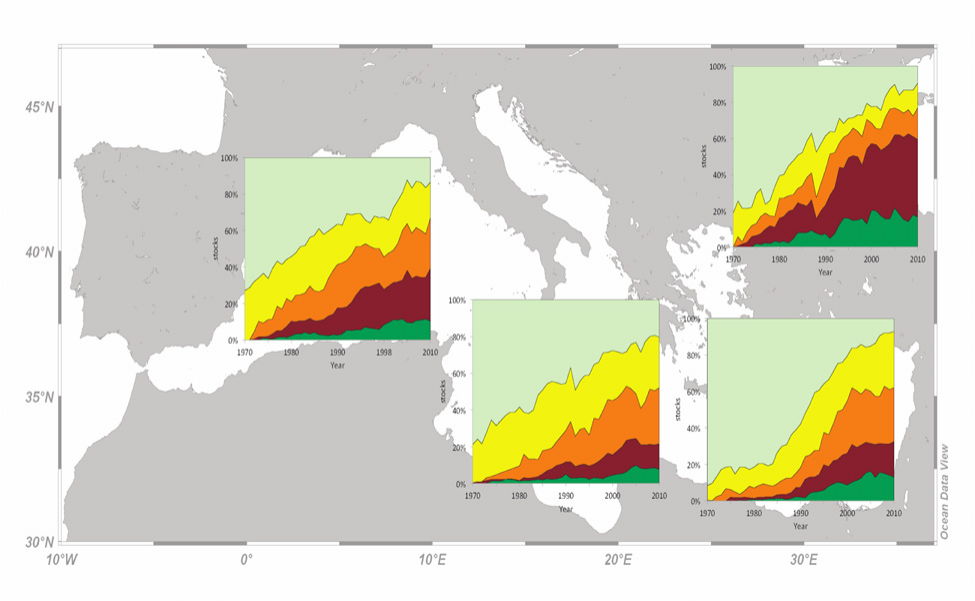
The trend for all stocks according to the catch-based method, for the western, central, eastern Mediterranean fishing subarea and the Black Sea for the period 1970 to 2010 (light green: developing; yellow: fully exploited; orange: overexploited; brown: collapsed; dark green: recovering).

The percentage of developing stocks declined linearly with time across subareas, while, at the same time, the cumulative percentage of overexploited and collapsed stocks increased linearly (Fig. 7). The higher decline in developing stocks was observed in the E Mediterranean and the lower decline in the W and C parts of the sea. Indeed, the rate of decline in developing stocks was 21, 20, 54 stocks per decade in the W, C, E Mediterranean, respectively, and 30 stocks per decade in the Black Sea. The higher increase in the cumulative percentage of overexploited and collapsed stocks was observed in the W and C Mediterranean and the lower increase in the Black Sea (Fig. 7). The rate of increase in overexploited and collapsed combined stocks was 86, 76, 62 stocks per decade for the W, C, E Mediterranean, respectively, and 46 stocks per decade for the Black Sea.

**Fig 8:**
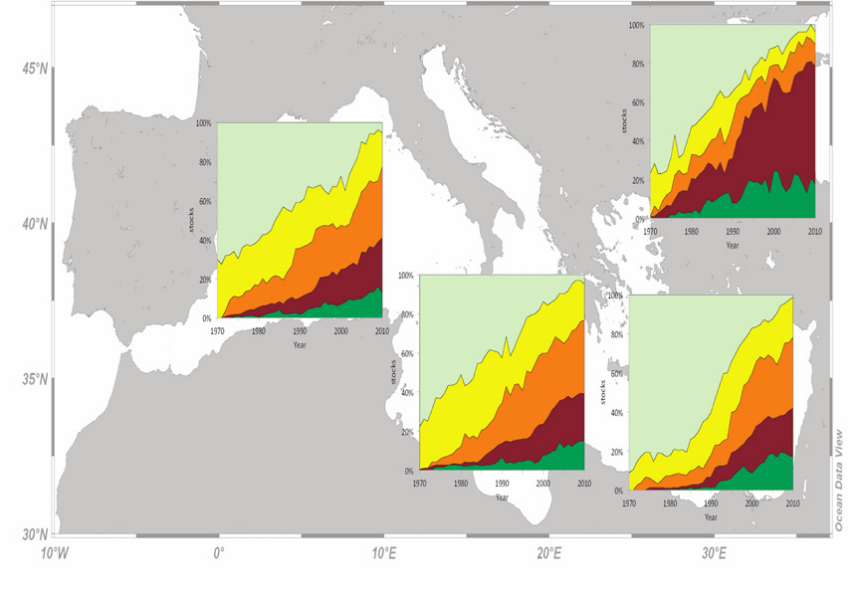
The trend considering only the stocks with 40 consecutive records according to the catch-based method, for the western, central, eastern Mediterranean fishing subarea and the Black Sea for the period 1970 to 2010 (light green: developing; yellow: fully exploited; orange: overexploited; brown: collapsed; dark green: recovering).

When only stocks with more than 40 consecutive records were considered, the cumulative percentage of overexploited and collapsed stocks in 2010 increased across subareas. It was 72% for the Black Sea, 65% for the W Mediterranean, 62% for the C Mediterranean and 61% for the E Mediterranean (Fig. 8). The cumulative percentage of developing and recovering stocks was rather stable among subareas: 22% for the Black Sea, 18% for the W Mediterranean, 19% for the C Mediterranean and 18% for the E Mediterranean (Fig. 8). Developing stocks were declining at a rate of 25, 36, 44 stocks per decade for the W, C, E Mediterranean, respectively, and 21 stocks per decade for the Black Sea. Overexploited and collapsed stocks were increasing at a rate of 44, 33, 38 stocks per decade for the W, C, E Mediterranean, respectively, and 13 stocks per decade for the Black Sea.

The exploitation pattern differed among the subdivisions of each subarea of the Mediterranean and the Black Sea (Table 2). In 2010, the cumulative percentage of overexploited and collapsed stocks exceeded 50% in all fishing subdivisions, except subdivisions 2.1 (Adriatic) and 3.2 (Levantine), which presented the lowest cumulative percentage of overexploited and collapsed stocks (Table 2). The degree of exploitation also varied among subdivisions. Subdivisions 1.2 (Lions Gulf), 3.1 (Aegean Sea), 4.1 (Marmara Sea) and 4.2 (main Black Sea) were the most heavily exploited ones with the cumulative percentage of overexploited and collapsed stocks exceeding 55% (Table 2). The highest percentage of developing fisheries stocks were recorded in subdivision 2.1 (Adriatic) and the highest percentage of recovering stocks in subdivisions 4.2 (main Black Sea), 4.1 (Marmara) and 3.2 (Levantine) (Table 2).

**Table 2:** The status in 2010 of fisheries resources according to the catch-based method per GFCM fishing subdivision (area codes as they appear on Fig. 1) of the Mediterranean and Black Sea.

Exploitation	Developing Number (%)	Fully exploited Number (%)	Overexploited Number (%)	Collapsed Number (%)	Recovering Number (%)	Total stocks
Area						
**Mediterranean Sea**						
Balearic (1.1)	25 (11)	67 (30)	69 (31)	44 (20)	18 (8)	**223**
Gulf of Lions (1.2)	26 (15)	19 (11)	34 (20)	70 (42)	20 (12)	**169**
Sardinia (1.3)	35 (17)	37 (18)	64 (32)	45 (22)	22 (11)	**203**
Adriatic (2.1)	52 (26)	53 (27)	48 (24)	24 (12)	22 (11)	**199**
Ionian (2.2)	63 (18)	94 (26)	125 (35)	54 (15)	21 (6)	**357**
Aegean (3.1)	4 (3)	32 (25)	52 (40)	28 (22)	13 (10)	**129**
Levantine (3.2)	24 (11)	69 (30)	59 (26)	40 (18)	33 (15)	**225**
**Black Sea**						
Marmara (4.1)	0 (0)	4 (8)	12 (25)	24 (50)	8 (17)	**48**
main Black Sea (4.2)	16 (14)	13 (11)	17 (14)	49 (42)	23 (19)	**118**
Azov Sea (4.3)	5 (14)	11 (31)	7 (19)	11 (31)	2 (5)	**36**
**Total**						**1707**

### Comparison of the catch-based method with stock assessment data

**Fig 9:**
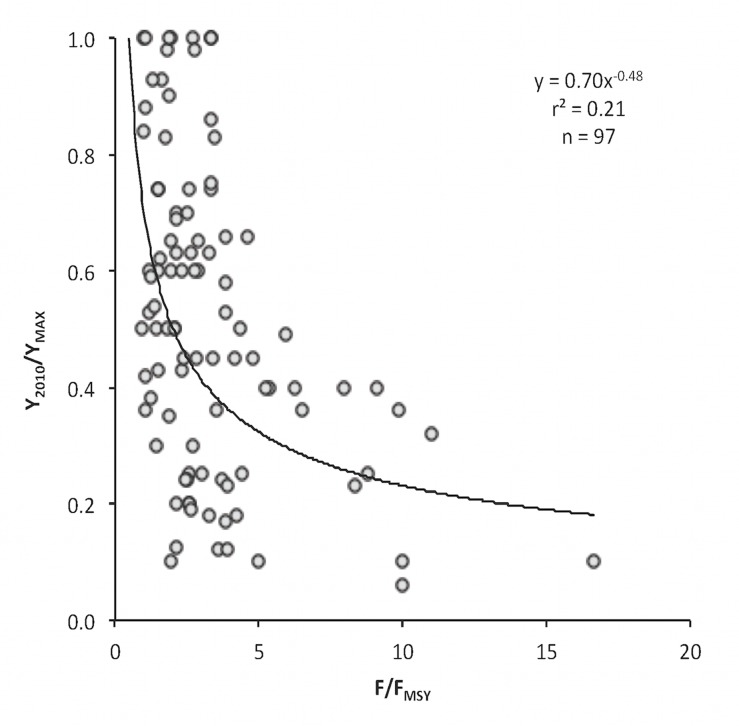
The relationship of the ratio of the current fishing mortality (F) and mortality at MSY (F_MSY_) with the ratio of the latest catch in 2010 (Y_2010_) to historically maximum catch (Y_MAX_) for 97 stocks for which both ratios were available (for explanation see maintext).

**Fig 10:**
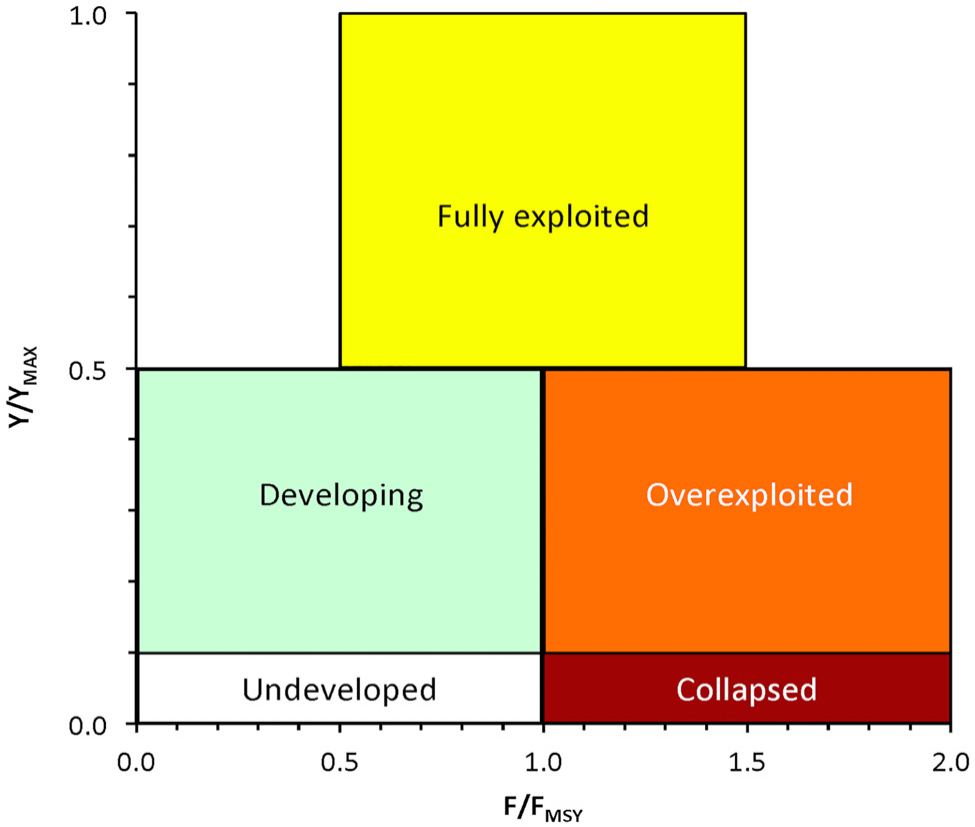
Theoretical plot of the stock status definitions (as they appear on Table 1, but excluding “recovering” for simplicity purposes) based on the relationship between the ratio of the latest catch (Y) to historically maximum catch (Y_MAX_) and their relationship with the ratio of current fishing mortality (F) to mortality at MSY (F_MSY_).

The relationship between the F/F_MSY_ ratio and the Y_2010_/Y_MAX_ ratio was expressed by an inverse power function (Fig. 9), which shows that high fishing mortality rates may lead to low yields and stock collapses. According to the theoretical plot of this relationship (Fig. 10, *sensu* [50,51]), out of the 97 stocks from the entire Mediterranean Sea that were analyzed (Fig. 9), 44 stocks (45%) were fully exploited, 48 (49%) were overexploited, and 5 stocks (6%) were collapsed. No undeveloped/developing stocks were recorded (data not shown).

## Discussion

The results of the present work confirm, through the use of various indicators, that the fisheries resources of the Mediterranean and Black Sea are at risk from overexploitation. Similar conclusions on the overexploitation of these areas have been recently reported based on various datasets and approaches [5,6,19,52]. According to all indicators that were explored here, as summarized in Table 3, the pattern of exploitation and the state of stocks differs among subareas. All indicators confirmed the worst shape of the E Mediterranean and Black Sea fisheries compared to the W and C Mediterranean ones, none of which is underexploited. Indeed, in the E Mediterranean and the Black Sea, total catches, mTLc and FiB are declining, the cumulative percentage of overexploited and collapsed stocks exceeded 50% and developing stocks were less than 10%. In contrast, in the W Mediterranean, total catches are rather stable, mTLc and FiB are increasing following a long decline, and developing stocks exceed 10%. Finally, in the C Mediterranean, mTLc and FiB are also increasing following a long decline, the cumulative percentage of overexploited and collapsed stocks do not exceed 50% and developing stocks are 20%.

**Table 3:** Overview of all indicators (TC: total catch; CBM: catch-based method; mTLc: mean trophic level of the catch; FiB: fishing in balance index) used in the present work for evaluating the status of Mediterranean and Black Sea fisheries per fishing subarea, indicating their condition in 2010.

Indicator	TC	CBM	mTLc	FiB	Overall
Area					
**Mediterranean Sea**					
Western	Average	Bad	Average	Average	**Average**
Central	Bad	Average	Average	Average	**Average**
Eastern	Bad	Bad	Bad	Bad	**Bad**
**Black Sea**					
Black Sea	Bad	Bad	Bad	Bad	**Bad**

Good condition of a time-series refers to increasing, bad to declining and average to stable trends.

The number of records increased in all subareas as a result of the disaggregation and the finer taxonomic resolution in catch records, partly because of the EU Fisheries Data Collection Framework that is applied to the European countries of the northern Mediterranean coastline. The higher number of records in the W and C Mediterranean compared to the E one (Fig. 2) agrees with the general biodiversity pattern in the Mediterranean Sea and reflects the number of species, which declines from northwestern to southeastern regions following the primary production gradient [35]. Specifically, the number of fish species that form the majority of catches decreases from western to eastern regions and is generally higher along the Mediterranean coastline [35].

Given that global fishing effort remains the same or increases with time, as it also does in the Mediterranean Sea [53], a decline in total catches, which is actually a decline in catch per unit of effort (CPUE), can be considered as a suspicion or indication of overfishing. There are a few well-documented examples where CPUE successfully tracks the stock size [54] but this is not always the case. In cases where fishing mortality follows predictable dynamics over time, catch data can be used to reconstruct biomass and potentially used for conducting data-poor assessments [55]. Declines in CPUE have been reported for the vast majority of FAO areas, including the Mediterranean Sea, where a drastic CPUE decline is apparent since the late 1990s [1]. The more or less stable total catches of the W Mediterranean, but with a declining trend during the last decade, is probably the result of exploiting new species, as revealed by the number of added records per year (Fig. 2), and to improving management practices as revealed by the larger number of stocks that are regularly assessed [9]. The declining trend of C Mediterranean total catches after mid 1980s and of the E Mediterranean ones after mid 1990s (Fig. 3), is a clear effect of excessive fishing mortality applied on these stocks [42] and the alteration and simplification of the food web structure over time as a result of fishing [35,56]. Overfishing and its side effects on ecosystem have been blamed for the collapse of Black Sea fisheries in early 1990s and the decline in total catches [28]. Some catch fluctuations, however, especially those of the small and medium pelagic fishes that are highly represented in the catches, could be the result of climatic effects [57] or modifications in the planktonic production [58].

The trends of mTLc were declining for all subareas until about 2005, when they started to increase again for the W and C Mediterranean (Fig. 4). The abrupt increase in the mTLc in the W Mediterranean after 2005 (Fig. 4) may be attributed to either a decline in the catches of low trophic level species, or an increase in the catches of high trophic level species. In any case, this is a clear situation of fishing-up the marine food webs [8]. This mTLc increase, together with a simultaneous decline in catches, produced a rather stable FiB trend with a slight increasing tendency during the last decade (Fig. 5). Since 2007, it seems that at least one ecosystem of the W Mediterranean (Gulf of Lions) has shifted to a different regime, which is characterized by low sardine and anchovy catches [58]. According to the same work [58], the landings of sardine and anchovy declined by 50% and 70%, respectively, which confirms the fishing-up effect of the last lustrum. Similarly, a decline in top predators and large sized fish, as well as an increase of lower trophic level species has been reported for another ecosystem of the W Mediterranean, the Catalan Sea [56]. The higher exploitation rate of demersal and large pelagic stocks in the Adriatic Sea since 1990 has lead to mTLc decline until about 2005, when mTLc started to increase again (Fig. 4). The exact same pattern was apparent in FiB (Fig. 5), which is a function of the mTLc, when catches are stable or follow the same trend. Similarly to the W Mediterranean, a long-term decline in large-sized, high trophic level species has been reported to occur during the same period for the Adriatic Sea [59], together with an increase in species positioned lower in the food web [60]. After 2005, the situation seems reversed, a pattern that was apparent in the FiB values as well. In the E Mediterranean Sea, the decline in mTLc (Fig. 4), albeit at low rates compared to other areas, was continuous. Because of the low rate of mTLc decrease, FiB followed the fluctuation of catches [38]. A decline in mTLc for species with trophic level greater than 3.75 has been shown to occur for the Greek Seas [61], whereas stable mTLc values for all species have been recently reported for the same area [7]. Based on the latter, the decline in mTLc is more likely to occur because the Aegean Sea is being exploited both by the Greek and Turkish fishing fleets. Indeed, the trawling and dredging disturbance as well as the exploitation pattern is very high along the Aegean coastline and lower in the Levantine Sea [62], where the stocks are generally in a better shape (Table 2). The collapse of the Black Sea fisheries in 1990 and the decline in the mTLc since then, is apparent in the FiB values that declined accordingly (Figs. 4 and 5). Indeed, a decrease in abundance of high trophic level species and an increase of low trophic level species has been reported to occur after 1990 for the Black Sea [63]. One of the reasons for the collapse of fisheries in the Black Sea was the invasion of the ctenophore *Mnemiopsis leidyi*, which preys upon the eggs and larvae of pelagic fish and may cause population declines [64].

Regarding the status of exploitation according to the catch-based method (Figs. 6 and 7), a few points can be considered encouraging compared to the current global pattern. Firstly, the percentage of developing stocks, and hence developing fisheries, is generally higher in all Mediterranean subareas and, secondly, the cumulative percentage of overexploited and collapsed ones is lower, when compared to an analysis on global fisheries [65] and the northeast Atlantic [51]. Developing stocks indicate the exploration of new fisheries and exploitation of new stocks or increasing stock biomass that is reflected upon catches, whereas recovering stocks indicate good or improving management practices applied on fisheries that had been previously depleted or collapsed [26]; but see also [66]. In the Adriatic Sea (C Mediterranean) the vast majority of demersal and large pelagic stocks had been classified as overfished since 2000, with the small and medium pelagic ones being in a better state [60]. In the Aegean Sea (E Mediterranean), about 65% of the Greek stocks were characterised as overexploited and 32% as fully exploited in 2007 [7], whereas the 85% of the Black Sea stocks are overexploited [29].

The use of stocks with consecutive records may limit the number of stocks included in the analysis but shows the fate of the catches of the same community through time and it is far less biased by the number of new records added each year [5]. When new species are added separately in the analysis (after disaggregation), they are by definition developing. Therefore, the percentage of developing stocks is being artificially increased and, at the same time, the percentage of the remaining categories proportionally decreased [7]. This bias is eliminated only when stocks with non-consecutive records are excluded and for that reason we re-analyzed the dataset including only stocks with consecutive records (Fig. 8). Once this effect was removed, the cumulative percentage of overexploited and collapsed stocks increased across subareas and that of the developing ones decreased (Fig. 8). The effect was more intense in W and C Mediterranean, where the number of stocks added each year was higher. This clearly indicates that the addition of new records may mask the true condition of stocks by increasing the percentage of developing ones [5]. The rate of increase of overexploited and collapsed consecutive stocks in the Black Sea was lower compared to other subareas because the actual number of stocks with consecutive records was very low (n = 78) compared to the non-consecutive ones (n = 283).

Finally, when the data from stock assessments was assembled and compared to the catch-based method (Fig. 9), the pattern of the relationship between the F/F_MSY_ and the Y_2010_/Y_MAX_ was similar to the F/F_MSY_ against B/B_MSY_ that has been recently published [50]. A comparison of Y_2010_/Y_MAX_ with B/B_MSY_ would have been preferable because catch-based status predictions refer to biomass and not fishing mortality. However, B/B_MSY_ values are not as commonly used in the Mediterranean stock assessments and are more biased compared to F/F_MSY_ estimates. The results of this relationship (according to Fig. 10), i.e., that the majority of recently assessed stocks are either fully exploited or overexploited, imply that assessments are applied to stocks for which there is suspicion or indication for overexploitation. When not routinely performed, as it is the case for most Mediterranean species, stock assessments can be considered as snapshots of the stock state that may not necessarily reflect its true condition. The catch-based method may have some disadvantages related to the quality of data [50], but it provides a time series of the condition of all stocks in one area and can be used as a first indicator of ecosystem health or its response to fishing [26]. It should be noted here that, in general, trophodynamic indicators are considered rather conservative indicators of ecosystem health because they tend to respond slowly to structural ecosystem changes [38].

The findings of the present work, through the use of various indicators, clearly show that the Mediterranean and Black Sea fisheries resources are at risk from overexploitation and that the degree of exploitation varies among subareas. They also sound the alarm bell for the remaining stocks and warn that cases of mismanagement should be abandoned [67]. Detailed and extensive stock assessments are required in relation to stock status and fishing mortality reference points, that will eventually lead to conservation policy through management measures.
